# The First Eight Mitogenomes of Leaf-Mining *Dactylispa* Beetles (Coleoptera: Chrysomelidae: Cassidinae) Shed New Light on Subgenus Relationships

**DOI:** 10.3390/insects12111005

**Published:** 2021-11-08

**Authors:** Shengdi Zhang, Lukáš Sekerka, Chengqing Liao, Chengpeng Long, Jiasheng Xu, Xiaohua Dai, Qingyun Guo

**Affiliations:** 1 Leafminer Group, School of Life Sciences, Gannan Normal University, Ganzhou 341000, China; 18645133878@163.com (S.Z.); chqdyx@126.com (C.L.); lchp617650632@gmail.com (C.L.); xjstis@163.com (J.X.); 2 National Navel-Orange Engineering Research Center, Ganzhou 341000, China; 3 Department of Entomology, National Museum, Natural History Museum, 1740 Cirkusová, Czech Republic; sagrinae@gmail.com; 4 College of Plant Protection, Hunan Agricultural University, Changsha 410128, China

**Keywords:** *Triplispa*, *Platypriella*, mitochondrial genome, phylogenetic tree, host plant, body shape

## Abstract

**Simple Summary:**

With 387 species, *Dactylispa* is a large genus of family Chrysomelidae, subfamily Cassidinae, and the most species-rich genus of leaf-mining hispine beetles. Among leaf-mining hispines, *Dactylispa* also feeds on the largest number of host plants, including 29 families and 80 genera, with the main hosts belonging to Poaceae, Rosaceae, and Fagaceae. Some species of *Dactylispa* are economic crop pests. However, the subgenus classification of *Dactylispa* species, which is currently based on morphology only, is problematic. Molecular phylogenetic studies may help solve this problem. In this study, we aimed to report the first eight mitochondrial genomes of *Dactylispa* and construct the first molecular phylogenetic trees of *Dactylispa*. The evolutionary relationships among three subgenera of *Dactylispa* were partially resolved by the molecular trees. However, more species should be included to solve the evolutionary issues for this genus of leaf-mining beetles.

**Abstract:**

The taxonomic classification of *Dactylispa*, a large genus of leaf-mining beetles, is problematic because it is currently based on morphology alone. Here, the first eight mitochondrial genomes of *Dactylispa* species, which were used to construct the first molecular phylogenies of this genus, are reported. The lengths of the eight mitogenomes range from 17,189 bp to 20,363 bp. All of the mitochondrial genomes include 13 protein-coding genes (PCGs), 22 transfer RNA genes (tRNAs), 2 ribosomal RNA genes (rRNAs), and 1 A + T-rich region. According to the nonsynonymous/synonymous mutation ratio (Ka/Ks) of all PCGs, the highest and the lowest evolutionary rates were found for atp8 and cox1, respectively, which is a common phenomenon among animals. According to relative synonymous codon usage, UUA(L) has the highest frequency. With two Gonophorini species as the outgroup, mitogenome-based phylogenetic trees of the eight *Dactylispa* species were constructed using maximum likelihood (ML) and Bayesian inference (BI) methods based on the PCGs, tRNAs, and rRNAs. Two DNA-based phylogenomic inferences and one protein-based phylogenomic inference support the delimitation of the subgenera *Dactylispa s. str*. and *Platypriella* as proposed in the system of Chen et al. (1986). However, the subgenus *Triplispa* is not recovered as monophyletic. The placement of *Triplispa* species requires further verification and testing with more species. We also found that both adult body shape and host plant relationship might explain the subgeneric relationships among *Dactylispa* beetles to a certain degree.

## 1. Introduction

With 387 species, *Dactylispa* Weise is a large genus of leaf-mining hispine beetles in Chrysomelidae [1,2,3,4,5]. *Dactylispa* also feeds on more host plant species than any other leaf-mining hispine genus, including plants from 29 families (mainly Poaceae, Rosaceae, and Fagaceae) [2,3]. Some species of *Dactylispa* are economic crop pests: *D. setifera* Chapuis and *D. spinosa* Weber on corn [1,4]; *D. lenta* Weise, *D. bayoni* Gestro, and *D. spinigera* Gyllenhal on rice [4,6]; and *D. dohertyi* Gestro on apple trees [7]. *Dactylispa* is widely distributed in the tropical and subtropical areas of the Old World, while a few species can reach temperate regions in northern China, the Russian Far East, and Japan [1,4]. With rich species, diverse hosts, and a wide distribution, *Dactylispa* spp. present high inter/intraspecific morphological variations in response to different environments. Thus, some *Dactylispa* species are not easy to identify based on morphological characteristics alone [1]. Some researchers have attempted to construct internal phylogenetic relationships within *Dactylispa* [1,8,9]. For example, Maulik’s (1919) system classifies Burmese and Indian species into six groups based on the number of spines; it is helpful for identification but not compatible with actual phylogenetic relationships [8]. Uhmann’s (1954) system is also purely based on morphological characteristics; moreover, he treated the African and Oriental species separately [9]. Chen’s (1960) system divides Chinese species into three subgenera, i.e., *Dactylispa s. str*., *Triplispa*, and *Platypriella*; the differences among them are somewhat apparent, but some intermediate forms still exist [1].

In Chen’s system, the nominotypical subgenus *Dactylispa s. str*. is distinguished by lateral margins of elytra that are not explanate, by punctures outside interstice VIII arranged in a single row in the middle but doubled at the base and apex, and by a prothorax with generally two to three lateral spines. The subgenus *Triplispa* is distinguished by lateral margins of elytra equal or almost equal in width throughout, the absence of minute conical tubercles on its upper surface, and punctures outside interstice VIII generally arranged in two rows; in contrast, the subgenus *Platypriella* is distinguished by explanate lateral margins of elytra that are strongly broadened at the base, and sometimes also at the apex, with several minute conical tubercles on the surface, and by punctures outside interstice VIII arranged in two rows in the middle but three rows at the base and apex [1]. The numbers of Chinese species in the above three subgenera are 41, 38, and 10, respectively [1]. According to observations of limited specimens, the pupal morphologies of the three subgenera exhibit some differences, but the differences are minor [2].

The faunae of individual zoogeographical regions are more or less distinct, and most of the authors working on hispines simply ignored the fact that there are some possible characteristics for separating the individual groups into subgenera or seemingly essentially ignored any complex classification of this genus. Chen’s (1960) system, although quite limited and uniform, works to some extent when applied in the Oriental Region. *Triplispa*–*Platypriella* is complicated, and some taxa are challenging to place correctly [1]. Chen’s system, however, cannot be applied to species in Africa and Madagascar, many of which can be classified within *Dactylispa s. str*.; many of the others would more or less belong to *Triplispa,* but there is a substantial portion of species that cannot be placed within any subgenus. Some researchers will follow Chen’s system because it is practical and can be used for continental Asiatic *Dactylispa*; however, only future phylogenetic studies might solve this for good (LS, unpubl. data).

Insect mitogenomes are typically closed circular double-stranded DNA molecules with lengths ranging from 14 kb to 36 kb [10]. They usually contain 37 coding genes, including 13 protein-coding genes (PCGs), 22 transfer RNA genes (tRNAs) and 2 ribosomal RNA genes (rRNAs) [11,12]. They also have a long noncoding control region (i.e., the A + T-rich region), which plays an important role in the regulation of transcription and replication [12,13,14]. Mitochondrial genomes have many advantages in evolutionary analyses, including a small size, a high copy number, a wide range of evolutionary rates, maternal inheritance, a lack of recombination and high mutation rates [15,16,17,18]. Therefore, mitogenomes have been widely adopted in molecular phylogenetics, evolutionary ecology, population genetics and phylogeography [11,19]. Moreover, combinations of the above 37 genes in a mitogenome might improve phylogenetic resolution [19].

Generally, there are only a few publications on *Dactylispa*, especially in recent years. For example, in a search for “allintitle: Dactylispa” on Google Scholar, there are only 33 hits, with only one article since 2015. Most recent publications focus on morphological descriptions, biological notes, and economic importance [6,7,20,21,22,23,24], without enough attention devoted to phylogenetic relationships. There are no studies on the mitogenomes and molecular phylogeny of *Dactylispa*. In this study, we aim to report the first eight mitochondrial genomes of *Dactylispa* and construct the first molecular phylogeny of *Dactylispa* to explore the evolutionary relationships among the three subgenera (*Dactylispa s. str.*, *Triplispa*, and *Platypriella*).

## 2. Materials and Methods

### 2.1. Sampling and DNA Extraction

The specimens were collected from the following places: Xiangshan, Xunwu County, Jiangxi Province (24°56′12.58″ N, 115°48′41.61″ E); Anjishan, Longnan County, Jiangxi Province (24°52′23.54″ N, 114°36′10.04″ E); and Taizhong Town, Jingdong County, Yunnan Province (24°27′0.11″ N, 100°49′57.89″ E). According to morphological characteristics, the specimens were identified as *D. approximate* Gressitt, *D. albopilosa* Gestro, *D. longispina* Gressitt, *D. planispina* Gressitt, *D. latispina* Gestro, *D. nigrodiscalis* Gressitt, *D. paucispina* Gressitt, and *D. xanthopus* Gestro. They were all stored in the Nanling Herbarium, Gannan Normal University (GNNU). Adults were all stored in 100% ethanol at −80 ℃. Whole genomic DNA was obtained from the head tissue of a single specimen following the manufacturer’s protocol with the TIANamp Genomic DNA Kit (TianGen, Beijing, China). DNA was preserved at −20 ℃ and sent to Shanghai Personal Biotechnology Co., Ltd. (Shanghai, China), for mitogenome sequencing.

### 2.2. Genomic Sequencing, Assembly, and Annotation

Total mitogenomes were obtained by next-generation sequencing (NGS) with the whole-genome shotgun (WGS) strategy on the Illumina MiSeq platform [19]. The reads of sequences were cleaned to remove the adaptor sequences and low-quality reads with ambiguous sequences [19]. The high-quality second-generation sequences were de novo assembled to obtain contigs and scaffolds using A5-miseq v20150522 [25] and SPAdes v3.9.0 [26]. Sequences were extracted based on the sequencing depth, and the sequences with high sequencing depths were then identified by the NCBI NT library using BLASTN (BLAST v2.2.31+) [27]. MUMmer v3.1 [28] was used to perform collinearity analysis, confirm the contig positions, and fill the gaps between contigs. Pilon v1.18 [29] was applied to correct the results and obtain the final mitochondrial sequences. Each mitogenome was annotated using Geneious R11 software, and coding regions were found through ORFfinder (https://www.ncbi.nlm.nih.gov/orffinder/, accessed on 12 November 2020) and manually verified by the BLAST tool on the NCBI website. tRNA structures were predicted and determined by MITOS2 (http://mitos.bioinf.uni-leipzig.de/index.py, accessed on 12 November 2020). The rRNA gene boundaries were interpreted as the end of a bounding tRNA gene and the alignment of sequences with homologous regions of known coleopteran mitogenomes. Circular mitogenome maps were generated using the CG View server V 1.0 [30,31].

### 2.3. Bioinformatic Analysis

Geneious R11 was employed to calculate the A + T and G + C contents, as well as the AT skew and GC skew. Here, AT skew = (A − T)/(A + T) and GC skew = (G − C)/(G + C). DnaSP v5 [32] was used to calculate the nonsynonymous substitution rate to synonymous substitution rate (Ka/Ks) ratio, and PhyloSuite [33] was used to calculate the relative synonymous codon usage (RSCU) for PCG analysis. Annotated sequences of the eight mitogenomes of *Dactylispa* beetles mentioned above were deposited in GenBank with the accession numbers specified in Table 1. We used DnaSP [32] to calculate the nucleotide divergence of 13 PCGs, 22 tRNA genes and two tRNA genes among eight *Dactylispa* species and compared the nucleotide diversity among the three subgenera of *Dactylispa*.

### 2.4. Phylogenetic Analysis

Phylogenetic analyses were based on the concatenated nucleotide sequences of all 37 genes (13 PCGs, 22 tRNAs, and 2 rRNAs) from the mitogenomes of eight *Dactylispa* species and two Gonophorini (Chrysomelidae: Cassidinae) species (*Agonita chinensis* and *Downesia tarsata*) as the outgroup (Table 1).

Each mitogenomic gene was aligned in MAFFT [34] with the normal alignment mode for the 24 RNAs and with the codon alignment mode for the 13 PCGs. The code table was ‘5 Invertebrate mitochondrial’, and the strategy was ‘auto’. The multiple alignments were then concatenated into one FASTA file. The best partitioning scheme and evolutionary models for 37 predefined partitions were then selected using PartitionFinder2 [35] with the greedy algorithm and AICc criterion (File S1). Maximum likelihood (ML) inference was performed in IQ-TREE [36] with the optimal model of GTR + I + G for 1000 bootstrap replications. Bayesian inference (BI) was performed using MrBayes 3.2.6 [37] under the GTR + I + G + F model with 2 million MCMC generations and 2 parallel runs in which the initial 25% of sampled data were discarded as burn-in. All of the above phylogenetic analyses were performed step by step in PhyloSuite [33].

The amino acid sequences of eight *Dactylispa* species and two outgroup species were translated from the nucleotide sequences of the 13 PCGs. The sequences were extracted, aligned, and concatenated using the above procedures in PhyloSuite [33]. Using the model of CAT + GTR, phylo-Bayesian (PB) inference was performed in Phylobayes MPI [38,39] on XSEDE (1.8c) with default parameters in the CIPRES Science Gateway [40].

**Table 1:** List of taxa used for the phylogenetic analysis in this study.

Subgenus	Species	GenBank No.	Size (bp)	Total A + T %	AT% of All PCGs	References
*Dactylispa s. str.*	*Dactylispa approximata*	MN016958	18,896	75.6	75.5	This study
*Dactylispa s. str*.	*Dactylispa albopilosa*	MN016964	18,774	70.9	70.5	This study
*Dactylispa s. str*.	*Dactylispa longispina*	MN016959	20,363	71.2	70.6	This study
*Triplispa*	*Dactylispa paucispina*	MN016963	18,116	74.9	74.7	This study
*Triplispa*	*Dactylispa nigrodiscalis*	MN016961	17,189	75.3	75.1	This study
*Triplispa*	*Dactylispa xanthopus*	MN016966	19,581	75	74.8	This study
*Platypriella*	*Dactylispa planispina*	MN016960	18,530	73.9	73.5	This study
*Platypriella*	*Dactylispa latispina*	MN016962	17,963	74.9	74.5	This study
OUTGROUP	*Agnoita chinensis*	MF351622	17,866	79.1	78.4	[41]
OUTGROUP	*Downesia tarsata*	MW176089	18,557	78.6	76.3	[42]

## 3. Results

### 3.1. Genome Organization and Nucleotide Composition

**Figure 1:**
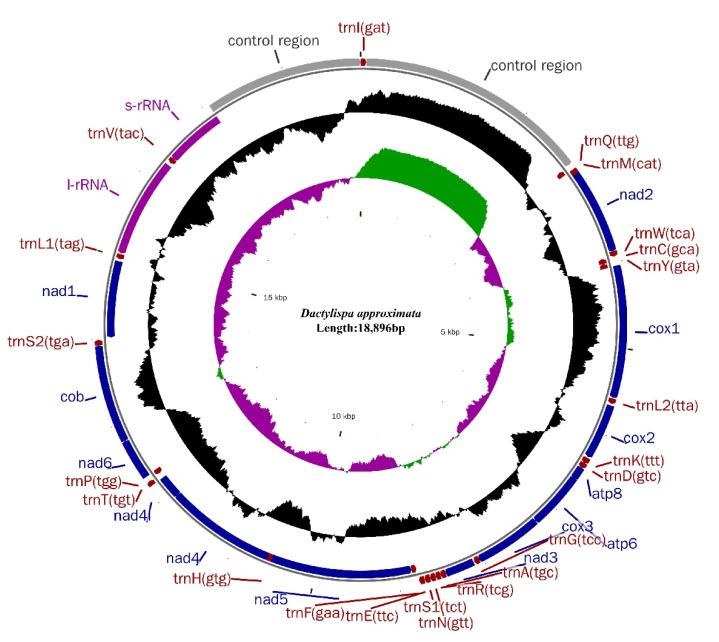
Circular diagram of the mitochondrial genome of *Dactylispa approximata*. Genes outside the circle are transcribed in a clockwise direction, whereas those inside the circle are transcribed counterclockwise. Protein-coding genes (PCGs) are in blue, tRNA genes are in red, and rRNA genes are in purple. The second circle shows the GC content, and the third circle shows the GC skew. The GC content and GC skew are plotted as the deviation from the average value of the entire sequence. Circular diagrams of the mitogenomes of the other seven *Dactylispa* species are shown in Figure S1.

The eight sequenced mitogenomes of *Dactylispa* species have lengths ranging from 17,189 bp to 20,363 bp (Table 1; Figure 1 and Figure S1). Each mitogenome contains the typical set of 37 genes (13 PCGs, 22 tRNAs, and 2 rRNAs) and an A + T-rich region (Table S1). In total, 23 genes (9 PCGs and 14 tRNAs) are located on the positive strand (N-strand); the other 14 genes (4 PCGs, 8 tRNAs and 2 rRNAs) are located on the reverse strand (J-strand) (Figure S1).

The nucleotide compositions of *Dactylispa* mitogenomes are biased toward A/T, with A + T contents ranging from 71.4% to 76.6%—values well within the range found in previously sequenced beetles (65.66–80.72%) [43]. Among the mitogenomes, those of *D. nigrodiscalis* and *D. albopilosa* exhibit the highest and lowest A + T contents, respectively. The AT skew and GC skew present similar patterns in all *Dactylispa* mitogenomes, with positive AT skews (from 0.093 to 0.154) and negative GC skews (from −0.262 to −0.110) (Table S2).

### 3.2. Protein-Coding Genes

The length of a single PCG varies from 153 bp (atp8) in all species to 1709 bp (nad5) in *D. latispina*. The total length of all 13 PCGs ranges from 10,959 bp in *D. approximata* to 10,998 bp in *D. paucispina* without stop codons (Table S1).

All 13 PCGs of *Dactylispa* species have typical initiation codons (ATN) and stop codons (TAR/TA/T). Complete termination codons (TAR) are generally used, except in the following cases: cox1, cox2, cox3, and nad5 in all *Dactylispa* species; nad4 in all *Dactylispa* species but *D. paucispina*; and nad3 and cytb in *D. xanthopus*. The incomplete stop codons (TA/T) may be converted to the complete stop codon (TAA) through RNA polyadenylation [31,44,45]. Incomplete stop codons are common in animal mitochondrial genomes [19,46,47].

**Figure 2:**
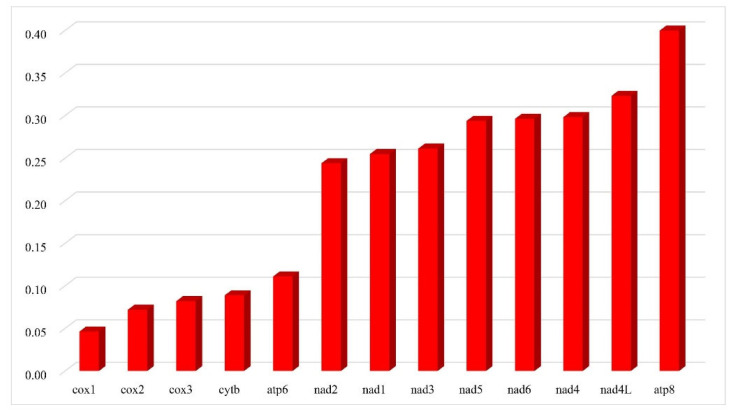
Ka/Ks ratios of 13 protein-coding genes. Ka is the nonsynonymous substitution rate, and Ks is the synonymous substitution rate.

The A + T content of the 13 PCGs ranges from 70.6% to 75.5%. Among the species, *D. approximata* shows the highest A + T content, while *D. albopilosa* presents the lowest A + T content. The PCGs exhibit positive AT skews (from 0.107 to 0.144) and negative GC skews (from −0.254 to −0.212) (Table S2). Among the 13 PCGs of the eight *Dactylispa* species, cox1 has the lowest Ka/Ks ratio, while atp8 has the highest (Figure 2).

### 3.3. Codon Usage

The most frequently used codons in the eight *Dactylispa* species are UUA, AUU, UUU, AUA, and AAU; in addition, GCG, CGC, AGC, CCG, and UGC are rarely used. However, RSCU indicated strong codon usage and amino acid composition biases. The codon usage comparison for the amino acids indicates that the codon UCN (Ser) is more frequently used than the codon AGN. For Leu, the UUN codon is more frequently used than CUN.

### 3.4. tRNAs and rRNAs

**Figure 3:**
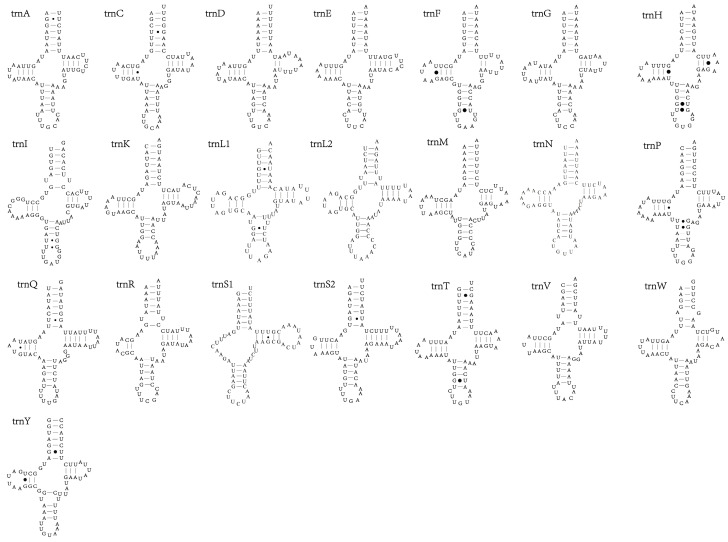
Secondary structure of 22 tRNAs identified in the mitogenome of *D. approximata*.

The 22 typical tRNAs were detected in all eight species (Figure 3), as in other beetles with published mitogenomes. All anticodons are highly conserved, as in other beetle species [48]. The total length of all tRNAs in the eight *Dactylispa* species mitogenomes range from 1331 bp to 1336 bp. Twenty-one tRNAs display the classic clover-leaf secondary structure, whereas trnS1 lacks the dihydrouridine (DHU) arm and forms a simple loop, which is common in other metazoan mitogenomes. Although somewhat less effective than conventional tRNAs, this abnormal tRNA is functional.

The A + T content of tRNAs ranges from 74.7% to 77.3%. Among the species, *D. approximata* and *D. latispina* exhibit the highest A + T content, while *D. albopilosa* presents the lowest A + T content. The tRNAs exhibit positive AT skews (from 0.048 to 0.100) and negative GC skews (from −0.154 to −0.080) (Table S2).

As in other insect mitogenomes, the 16S and 12S rRNA genes in *Dactylispa* species are encoded on the J-strand and located at conserved positions between trnL1 and trnV and between trnV and the control region, respectively [48]. The length of 16S ranges from 1035 bp in *D. longispina* to 1290 bp in *D. albopilosa*, and that of 12S varies from 719 bp in *D. planispina* to 793 bp in *D. nigrodiscalis*. The A + T content of 12S ranges from 75.50% in *D. longispina* to 79.20% in *D. approximata*. Hence, there is no substantial size variation in 12S among the mitogenomes of the eight *Dactylispa* species (Table S2).

### 3.5. Gene Overlap, Intergenic Spacers and Noncoding A + T-Rich Regions

**Figure 4:**
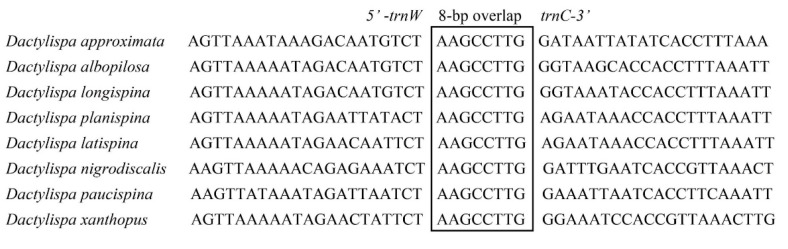
Sequence alignments of trnW/trnC of eight *Dactylispa* beetles. The boxed nucleotides are the eight-base pair conserved overlaps.

The size of overlapping regions was examined, varying from 1 bp to 24 bp in *D. albopilosa* and *D. planispina*, 1 bp to 22 bp in *D. latispina*, and 1 bp to 8 bp in the other five Dactylispa beetles. In all eight mitogenomes, 8 bp overlaps (AAGCCTTG) exist between tRNA-Trp and tRNA-Cys (Figure 4).

Furthermore, the size of intergenic spacers varies from 0 bp to 2692 bp in *D. approximata*; 0 bp to 1320 bp in *D. albopilosa*; 0 bp to 3049 bp in *D. longispina*; 0 bp to 1759 bp in *D. planispina*; 0 bp to 1099 bp in *D. latispina*; 0 bp to 327 bp in *D. nigrodiscalis*; 0 bp to 1358 bp in *D. paucispina*; and 0 bp to 2269 bp in *D. xanthopus*. In the eight *Dactylispa* mitogenomes, the longest intergenic spacers (327 bp to 3049 bp) are all situated between tRNA-Ile and tRNA-Gln.

The largest noncoding region in the mitogenomes of the eight *Dactylispa* beetles as well as most insects is usually located between rrnS and trnI. It might be identified as the putative control region according to the conserved region aligned with other Coleoptera mitogenomes [49]. The control regions of the eight *Dactylispa* mitogenomes vary widely, ranging in length from 1819 bp in *D. approximata* to 3069 bp in *D. albopilosa*, with an average A + T content of 77.61% (Table S2).

### 3.6. Genetic Diversity among Mitogenomes

**Figure 5:**
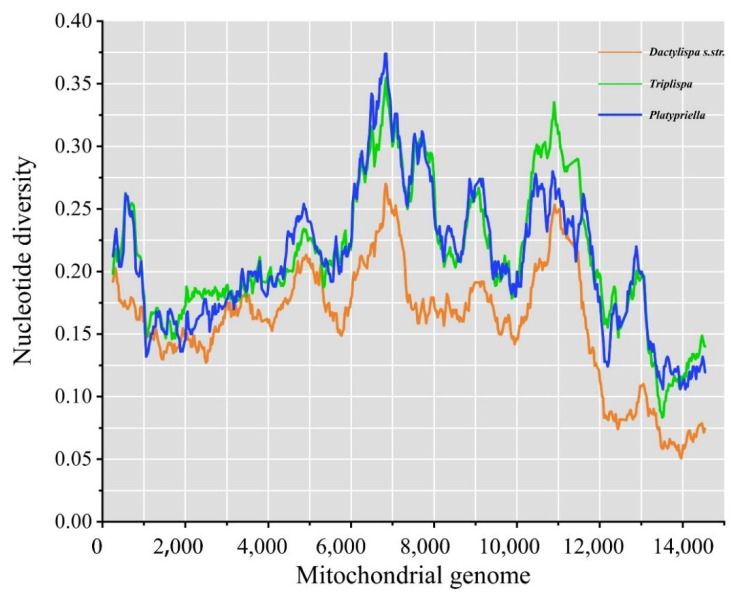
Sliding window analysis of 37 coding genes among 3 *Dactylispa* subgenera. The lines show the values of nucleotide diversity (Pi) in a sliding window analysis (a sliding window of 500 bp with a step size of 25 bp).

Mitogenome diversity shows similar trends among the three subgenera (*Dactylispa s. str.*, *Triplispa*, and *Platypriella*). In general, *Dactylispa s. str*. has the lowest nucleotide diversity, and *Triplispa* and *Platypriella* have similar patterns of nucleotide diversity (Figure 5).

### 3.7. Phylogenetic Analysis

**Figure 6:**
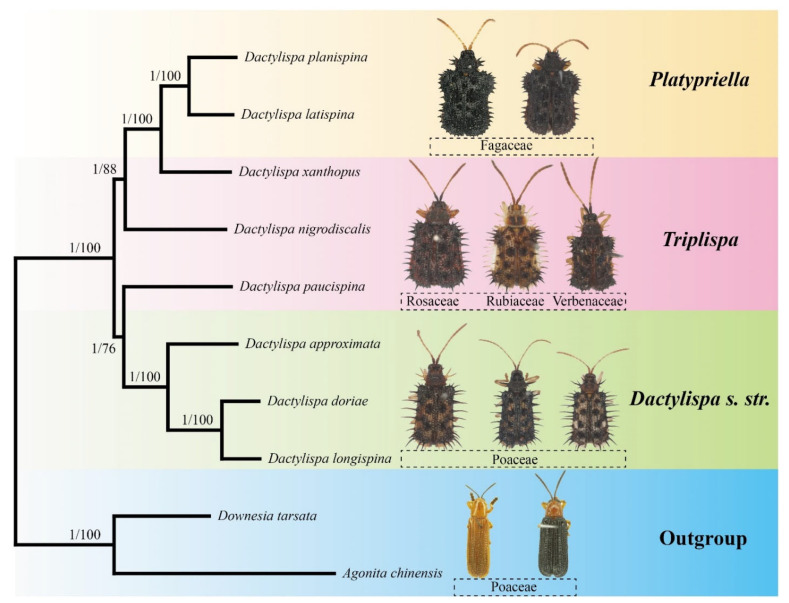
Phylogenetic tree of eight *Dactylispa* species based on complete PCGs, rRNAs, and tRNAs, with two Gonophorini species as the outgroup. Bootstrap values for the maximum likelihood (ML) analysis (**left**) and posterior probabilities for the Bayesian inference (BI) analysis (**right**) are shown for each node. Adults and their host plant families are also shown alongside the species names.

**Figure 7:**
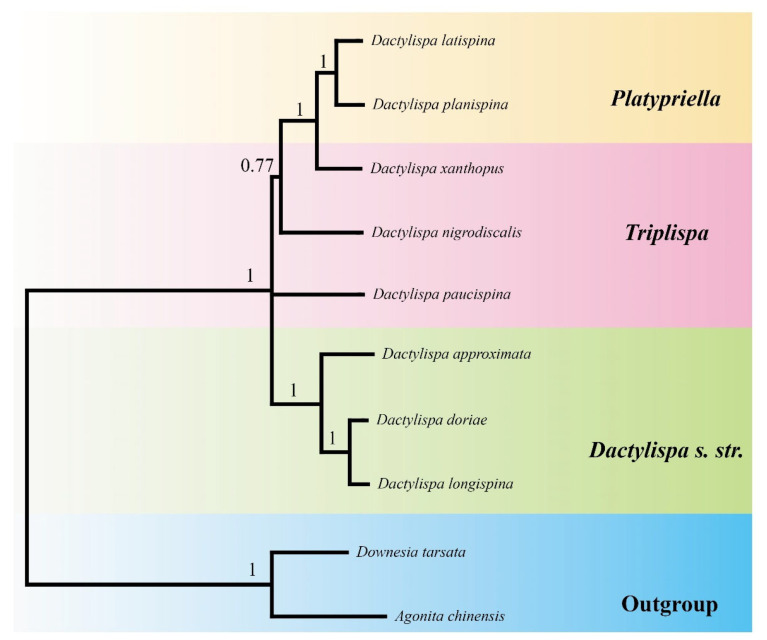
Phylogenetic tree of eight Dactylispa species based on complete PCG amino acids, with two Gonophorini species as the outgroup. Posterior probabilities for Bayesian inference are shown for each node.

Both ML and BI trees based on all mitochondrial genes (Figure 6) showed similar topologies. The genus *Dactylispa* was strongly supported as a monophyletic group (BP = 100, PP = 1), which was further divided into two major clades: the first clade includes the monophyletic subgenus *Dactylispa s. str*. (*D. approximata*, *D. albopilosa* and *D. longispina*) and the species *D. paucispina* belonging to *Triplispa* with high support in the BI tree (PP = 0.99) but moderate support in the ML tree (BP = 76). The second clade presented a nested form and consisted of the two *Triplispa* species (*D. xanthopuse* and *D. nigrodiscalis*) and the monophyletic subgenus *Platypriella* (*D. latispina* and *D. planispina*) with similar support as the former clade (BP = 88, PP = 1). In addition, the PB tree based on the amino acids of the 13 PCGs (Figure 7) was similar to the two nucleotide-based trees (Figure 6), except for the fact that *D. paucispina* was not included on the *Dactylispa s. str.* branch. The monophyly of both the subgenera *Dactylispa s. str*. and *Platypriella* was strongly supported by the phylogenetic trees, while the subgenus *Triplispa* appeared to be a paraphyletic group.

The *Dactylispa* species were separated according to their body shapes: slim species were on one branch, and broad species were on the other branch (Figure 6). Insect–host plant relationships might also help explain part of the *Dactylispa* phylogeny: all three species of *Dactylispa s. str*. are Poaceae-feeding, and both *Platypriella* species are Fagaceae-feeding, but the position of *Triplispa* could not be explained by host relationships alone [1].

## 4. Discussion

In this study, we sequenced and analyzed the first eight mitochondrial genomes of *Dactylispa* species. All of the sequenced *Dactylispa* mitogenomes exhibited the same gene arrangement, which was also consistent with the putative ancestral pattern in insects [50].

All *Dactylispa* sequences have the same 7 bp overlap “ATGATAA” between atp8 and atp6 and 8 bp overlap “AAGCCTTG” between trnW and trnC (Figure 4). Additionally, the atp6 and atp8 of the eight *Dactylispa* species have the same respective lengths of 663 bp and 153 bp. Similar intergenic spacers were also found in other Coleoptera, as well as in other insect orders [51]. Although these intergenic spacers have been hypothesized to be binding sites for translation termination [52], more research is still required to ascertain the functions of conserved noncoding regions in the mitogenomes of Coleoptera.

The Ka/Ks ratio is a powerful method of diagnosing the strength and mode of evolutionary selection on genes [53,54,55]. The Ka/Ks ratios for all PCGs of the eight *Dactylispa* species showed that cox1 had the lowest evolutionary rate, with atp8 having the highest (Figure 2). The cox1 gene was highly conserved in nearly all animals [19,56] and is thus the best DNA barcode in animal taxonomy and species phylogenetics [57]. The atp8 gene evolves rapidly in many animals [58,59], indicating that it is usually under weak selection pressure [19,60].

Our phylogenetic analysis indicates that the *Dactylispa* genus is separated into two clades, although these clades were strongly supported in the BI tree but only moderately supported in the ML tree (Figure 6). All three phylogenomic trees (Figure 6 and Figure 7) support the delimitation of the subgenera *Dactylispa s. str.* and *Platypriella* as proposed in Chen’s system [1]. However, *Triplispa* seems to be an invalid paraphyletic group. One reason is that Chen’s concept of *Triplispa* is broad, and he did not try to define any other subgenera because all “fit” within his concept [1]. In general, it is difficult to find true synapomorphies of *Dactylispa* as they have so many homoplasies that each having various states is not conservative. In theory, one can propose as many subgenera as they wish using peculiar characters. However, this is not a good approach in practice. In our study, not all species classified within the subgenus *Triplispa* belonged and thus rendered the subgenus poly- and paraphyletic. However, this does not contradict its validity; we simply do not have enough data to resolve this with certainty. 

The most interesting result is the separation of *D. paucispina* from the rest of the *Triplispa–Platypriella* clade (Figure 6 and Figure 7), rendering *Triplispa* polyphyletic. The entire *D. angulosa* group is odd looking, and our results indicate that it might (1) warrant its own subgenus or (2) belong to *Dactylispa s. str*. However, this branching has the lowest bootstrap value among the three that we are presenting (Figure 6 and Figure 7). Setting *D. paucispina* aside does not invalidate *Triplispa*, instead only showing that the phylogenetic history of the group is especially complicated. For the rest of *Triplispa–Platypriella*, there are at least two possible hypotheses: (1) the separation of *Platypriella* renders *Triplispa* paraphyletic; or (2) both are monophyletic with some other internal groups that would have to be separated. However, we lack enough data to make a clear decision, and our study is more of a first insight into the phylogeny of this genus.

According to the morphological characters of Chen’s system [1], the subgenus *Triplispa* has many intermediate or transitional characters between those of the other two subgenera. For example, the moderate body shape of *Triplispa* species is in transition from the slim shape of *Dactylispa s. str*. and the broad shape of *Platypriella* (Figure 6). Mitogenome nucleotide diversity indicated that the differences between *Triplispa* and *Platypriella* are small but the differences between the two subgenera and *Dactylispa s. str*. are large (Figure 5). Insect–host plant associations might somewhat explain the relationships of such subgenus (Figure 6). Most *Dactylispa s. str*. species are specialized on monocotyledonous plants such as Poaceae [1,2,3,4,5], the same as many primitive groups of hispine beetles [61,62]. In contrast, most *Triplispa* species and most *Platypriella* species feed on dicotyledonous plants. However, *Platypriella* seems to prefer Fagaceae plants, while *Triplispa* seems to prefer other dicotyledonous families [1,2,3,4,5]. 

In the inference of high-level phylogeny in Coleoptera, the PB tree with the heterogeneous-site model performs better than both BI and ML trees with homogeneous-site models [43], and the amino acid-based trees have a more reliable topology than the nucleotide-based trees [43]. In our case, all the three inferences (PB, BI, and ML) are very similar in terms of tree topology except that *D. paucispina* is clustered with the *Dactylispa s. str.* branch in both BI and ML trees (Figure 6) but not so in the PB tree (Figure 7). Both body shape and host plant relation support that *D. paucispina* should be isolated from the *Dactylispa* *s. str.* species. Compared to both BI and ML, PB might also infer better low-level phylogeny in Coleoptera such as the subgenus relationships among the *Dactylispa* genus. 

The mitogenome is a good resource for separating species that are extremely similar in morphology, such as *Dactylispa* spp. However, our sample is still rather small. In the future, more species should be included to solve the evolutionary issues in this genus of leaf-mining beetles.

## Data Availability

All the mitochondrial genome sequences were submitted to GenBank (accessions MN016958-MN016966, as in Table 1), and they will be accessible when the article is published.

## Supplementary Materials

The following are available online at https://www.mdpi.com/article/10.3390/insects12111005/s1. Figure S1: Circular diagrams of eight *Dactylispa* mitogenomes. Table S1: Summary of eight *Dactylispa* mitochondrial genomes. Table S2: Nucleotide composition of mitochondrial genomes of eight *Dactylispa* species. File S1: PartitionFinder output files.

## References

[1] Chen S.H., Yu P.Y., Sun C.H., T’an C.H., Zia Y. (1986). Fauna Sinica (Insecta: Coleoptera: Hispidae).

[2] Liao C. (2015). Diversity and Host Relationship of Leaf-Mining Hispine Beetle. Master’s Thesis.

[3] Liao C., Xu J., Dai X., Zhao X. (2015). Species diversity of leaf-mining hispines and of their host plants. Ecol. Sci..

[4] Staines C. Catalog of the Hispines of the World (Coleoptera: Chrysomelidae: Cassidinae). https://naturalhistory.si.edu/research/entomology/collections-overview/coleoptera/catalog-hispines-world.

[5] Santiago-Blay J.A. (2004). Leaf-mining chrysomelids. New Developments on the Biology of Chrysomelidae.

[6] Banwo O.O., Makundi R.H., Abdallah R.S., Mbapila J.C. (2001). First Report of Dactylispa lenta Weise (Coleoptera Chrysomelidae) as a Vector of Rice Yellow Mottle Virus. Acta Phytopathol. Entomol. Hung..

[7] Gupta R., Tara J.S., Chhetry M. (2012). Bionomics of *Dactylispa Dohertyi* (Gestro, 1897), a new pest of apple plantations (*Malus domestica* Borkh.) in Jammu Region of J & K, India. Munis Entomol. Zool..

[8] Maulik S. (1919). The Fauna of British India including Ceylon and Burma: Coleoptera: Chrysomelidae (Hispinae and Cassidinae).

[9] Uhmann E. (1954). LXV.—Hispinae aus dem Britischen Museum.—VIII. Teil. 156. Beitrag zur Kenntnis der Hispinae (Coleopt., Chrysom.). Ann. Mag. Nat. Hist..

[10] Boore J.L. (1999). Animal mitochondrial genomes. Nucleic Acids Res..

[11] Cameron S.L. (2014). Insect mitochondrial genomics: Implications for evolution and phylogeny. Annu. Rev. Entomol..

[12] Sayadi A., Immonen E., Tellgren-Roth C., Arnqvist G. (2017). The Evolution of Dark Matter in the Mitogenome of Seed Beetles. Genome Biol. Evol..

[13] Zhang D.-X., Szymura J.M., Hewitt G.M. (1995). Evolution and structural conservation of the control region of insect mitochondrial DNA. J. Mol. Evol..

[14] Zhang D.-X., Hewitt G.M. (1997). Insect Mitochondrial Control Region: A Review of its Structure, Evolution and Usefulness in Evolutionary Studies. Biochem. Syst. Ecol..

[15] Curole J.P., Kocher T.D. (1999). Mitogenomics: Digging deeper with complete mitochondrial genomes. Trends Ecol. Evol..

[16] DeSalle R., Schierwater B., Hadrys H. (2017). MtDNA: The small workhorse of evolutionary studies. Front. Biosci. Landmark.

[17] Wilson A.C., Cann R.L., Carr S.M., George M., Gyllensten U.B., Helm-Bychowski K.M., Higuchi R.G., Palumbi S.R., Prager E.M., Sage R.D. (1985). Mitochondrial DNA and two perspectives on evolutionary genetics. Biol. J. Linn. Soc..

[18] Saccone C., Gissi C., Reyes A., Larizza A., Pesole G. (2002). Mitochondrial DNA in metazoa: Degree of freedom in a frozen event. Genes.

[19] Xiao L., Zhang S., Long C., Guo Q., Xu J., Dai X., Wang J. (2019). Complete Mitogenome of a Leaf-Mining Buprestid Beetle, Trachys auricollis, and Its Phylogenetic Implications. Genes.

[20] Dai X., Xu J., Jiang Z. (2012). Bionomics of Dactylispa approximata on Lophatherum gracile. North. Hortic..

[21] Lee C. (2009). The taxonomic status of Dactylispa taiwana Takizawa, 1978 (Coleoptera: Chrysomelidae: Cassidinae). Genus. Int. J. Invertebr. Taxon..

[22] Wen L.U., Zhang Y., Huang C. (2003). Study on the economic threshold of Dactylispa setifera (Chapuis). J. Agric. Biol. Sci..

[23] Zheng X.L., Zhang Y.J., Wang Y.L., Dong Z.S., Da-Xing H.U., Wen L.U., University G. (2016). Observation on digestive system in Dactylispa setifera Chapuis(Coleoptera:Hispidae). J. South. Agric..

[24] Zaitsev Y.M. (2012). The immature stages of the leaf-beetle genus Dactylispa (Coleoptera, Chrysomelidae) from Vietnam. Entomol. Rev..

[25] Coil D., Jospin G., Darling A.E. (2014). A5-miseq: An updated pipeline to assemble microbial genomes from Illumina MiSeq data. Quant. Biol..

[26] Bankevich A., Nurk S., Antipov D., Gurevich A.A., Dvorkin M., Kulikov A.S., Lesin V.M., Nikolenko S.I., Pham S., Prjibelski A.D. (2012). SPAdes: A new genome assembly algorithm and its applications to single-cell sequencing. J. Comput. Biol..

[27] Coordinators N.R. (2018). Database resources of the National Center for Biotechnology Information. Nucleic Acids Res..

[28] Kurtz S., Phillippy A., Delcher A.L., Smoot M., Shumway M., Antonescu C., Salzberg S.L. (2004). Versatile and open software for comparing large genomes. Genome Biol..

[29] Walker B.J., Abeel T., Shea T., Priest M., Abouelliel A., Sakthikumar S., Cuomo C.A., Zeng Q., Wortman J., Young S.K. (2014). Pilon: An integrated tool for comprehensive microbial variant detection and genome assembly improvement. PLoS ONE.

[30] Grant J.R., Stothard P. (2008). The CGView Server: A comparative genomics tool for circular genomes. Nucleic Acids Res..

[31] Nabil-Fareed A., Petty N.K., Zakour N.L.B., Beatson S.A. (2011). BLAST Ring Image Generator (BRIG): Simple prokaryote genome comparisons. BMC Genom..

[32] Librado P., Rozas J. (2009). DnaSP v5: A software for comprehensive analysis of DNA polymorphism data. Bioinformatics.

[33] Zhang D., Gao F., Jakovlić I., Zou H., Zhang J., Li W.X., Wang G.T. (2020). PhyloSuite: An integrated and scalable desktop platform for streamlined molecular sequence data management and evolutionary phylogenetics studies. Mol. Ecol. Res..

[34] Katoh K., Standley D.M. (2013). MAFFT multiple sequence alignment software version 7: Improvements in performance and usability. Mol. Biol. Evol..

[35] Lanfear R., Frandsen P.B., Wright A.M., Senfeld T., Calcott B. (2017). PartitionFinder 2: New Methods for Selecting Partitioned Models of Evolution for Molecular and Morphological Phylogenetic Analyses. Mol. Biol. Evol..

[36] Nguyen L.T., Schmidt H.A., von Haeseler A., Minh B.Q. (2015). IQ-TREE: A fast and effective stochastic algorithm for estimating maximum-likelihood phylogenies. Mol. Biol. Evol..

[37] Ronquist F., Teslenko M., van der Mark P., Ayres D.L., Darling A., Hohna S., Larget B., Liu L., Suchard M.A., Huelsenbeck J.P. (2012). MrBayes 3.2: Efficient Bayesian phylogenetic inference and model choice across a large model space. Syst. Biol..

[38] Lartillot N., Rodrigue N., Stubbs D., Richer J. (2013). PhyloBayes MPI: Phylogenetic reconstruction with infinite mixtures of profiles in a parallel environment. Syst. Biol..

[39] Lartillot N., Lepage T., Blanquart S. (2009). PhyloBayes 3: A Bayesian software package for phylogenetic reconstruction and molecular dating. Bioinformatics.

[40] Miller M.A., Pfeiffer W., Schwartz T. Creating the CIPRES Science Gateway for inference of large phylogenetic trees. Proceedings of the 2010 Gateway Computing Environments Workshop (GCE).

[41] Guo Q., Xu J., Liao C., Dai X., Jiang X. (2017). Complete mitochondrial genome of a leaf-mining beetle, Agonita chinensis Weise (Coleoptera: Chrysomelidae). Mitochondrial DNA B Resour..

[42] Zhang S., Guo Q., Xu J., Wang X., Dai X. (2021). The complete mitochondrial genome of Downesia tarsata (Coleoptera: Chrysomelidae: Cassidinae). Mitochondrial DNA B Resour..

[43] Yuan M.L., Zhang Q.L., Zhang L., Guo Z.L., Liu Y.J., Shen Y.Y., Shao R. (2016). High-level phylogeny of the Coleoptera inferred with mitochondrial genome sequences. Mol. Phylogenet. Evol..

[44] Ojala D., Montoya J., Attardi G. (1981). tRNA punctuation model of RNA processing in human mitochondria. Nature.

[45] Wang H.L., Yang J., Boykin L.M., Zhao Q.Y., Li Q., Wang X.W., Liu S.S. (2013). The characteristics and expression profiles of the mitochondrial genome for the Mediterranean species of the Bemisia tabaci complex. BMC Genom..

[46] Boore J.L. (2001). Complete Mitochondrial Genome Sequence of the Polychaete Annelid Platynereis dumerilii. Mol. Biol. Evol..

[47] Chen S.C., Wang X.Q., Li P.W., Hu X., Wang J.J., Peng P. (2016). The Complete Mitochondrial Genome of Aleurocanthus camelliae: Insights into Gene Arrangement and Genome Organization within the Family Aleyrodidae. Int. J. Mol. Sci..

[48] Su T., Liang A. (2019). Comparative analysis of seven mitochondrial genomes of Phymatostetha (Hemiptera: Cercopidae) and phylogenetic implications. Int. J. Biol. Macromol..

[49] Ren L., Shang Y., Yang L., Shen X., Chen W., Wang Y., Cai J., Guo Y. (2019). Comparative analysis of mitochondrial genomes among four species of muscid flies (Diptera: Muscidae) and its phylogenetic implications. Int. J. Biol. Macromol..

[50] Hong M.Y., Jeong H.C., Kim M.J., Jeong H.U., Lee S.H., Kim I. (2009). Complete mitogenome sequence of the jewel beetle, Chrysochroa fulgidissima (Coleoptera: Buprestidae). Mitochondrial DNA.

[51] Amorim I.C., Melo A.S., Cruz G.A.d.S., Wallau G.d.L., Moura R.d.C.d. (2017). Dichotomius (Luederwaldtinia) schiffleri (Coleoptera: Scarabaeidae) mitochondrial genome and phylogenetic relationships within the superfamily Scarabaeoidea. Mitochondrial DNA Part B.

[52] Sheffield N.C., Song H., Cameron S.L., Whiting M.F. (2008). A comparative analysis of mitochondrial genomes in Coleoptera (Arthropoda: Insecta) and genome descriptions of six new beetles. Mol. Biol. Evol..

[53] Hurst L.D. (2002). The Ka/Ks ratio:diagnosing the form of sequence evolution. Trends Genet..

[54] Jeffares D.C., Tomiczek B., Sojo V., dos Reis M. (2015). A beginners guide to estimating the non-synonymous to synonymous rate ratio of all protein-coding genes in a genome. Methods Mol. Biol..

[55] Zhang Z., Li J., Zhao X.-Q., Wang J., Wong G.K.-S., Yu J. (2006). KaKs_Calculator: Calculating Ka and Ks Through Model Selection and Model Averaging. Genom. Proteom. Bioinform..

[56] Nie R.-E., Yang X.-K. (2014). Research progress in mitochondrial genomes of Coleoptera. Acta Biochim. Biophys. Sin..

[57] Hebert P.D., Ratnasingham S., de Waard J.R. (2003). Barcoding animal life: Cytochrome c oxidase subunit 1 divergences among closely related species. Proc. Biol. Sci..

[58] Oliveira D.C., Raychoudhury R., Lavrov D.V., Werren J.H. (2008). Rapidly evolving mitochondrial genome and directional selection in mitochondrial genes in the parasitic wasp nasonia (hymenoptera: Pteromalidae). Mol. Biol. Evol..

[59] Smietanka B., Burzynski A., Wenne R. (2010). Comparative genomics of marine mussels (*Mytilus* spp.) gender associated mtDNA: Rapidly evolving atp8. J. Mol. Evol..

[60] Shen X., Li X., Sha Z., Yan B., Xu Q. (2012). Complete mitochondrial genome of the Japanese snapping shrimp Alpheus japonicus (Crustacea: Decapoda: Caridea): Gene rearrangement and phylogeny within Caridea. Sci. China Life Sci..

[61] Chaboo C.S. (2007). Biology and phylogeny of the Cassidinae Gyllenhal sensu lato (tortoise and leaf-mining beetles) Coleoptera Chrysomelid. Bull. Am. Mus. Nat. Hist..

[62] Wilf P., Labandeira C.C., Kress W.J., Staines C.L., Windsor D.M., Allen A.L., Johnson K.R. (2000). Timing the radiations of leaf beetles: Hispines on gingers from latest cretaceous to recent. Science.

