# Supplementary material for: The First Eight Mitogenomes of Leaf-Mining Dactylispa Beetles (Coleoptera: Chrysomelidae: Cassidinae) Shed New Light on Subgenus Relationships
Source: Insects. 2021 Nov 8;12(11):1005. doi: 10.3390/insects12111005 (PMC8624545; doi:10.3390/insects12111005)
Supplement: Supplementary file 1 [file insects-12-01005-s001.zip › Figure S1 Circular diagrams of eight Dactylispa mitogenomes.pdf]

Figure S1 Circular diagram of eight *Dactylispa* mitogenomes. (A) *D.approximata* (B) *D. albopilosa* (C) *D.longispina* (D) *D. paucispina* (E) *D. nigrodiscalis* (F) *D. chinensis* (G) *D. planispina* (H) *D. latispina*. Genes outside the circle are transcribed in a clockwise direction, whereas those inside the circle transcribed counterclockwise. Protein-coding genes (PCGs) are in blue, tRNA genes are in red, and rRNA genes are in purple. The second circle shows the GC content, and the third shows the GC skew. The GC content and GC skew plotted as the deviation from the average value of the entire sequence.

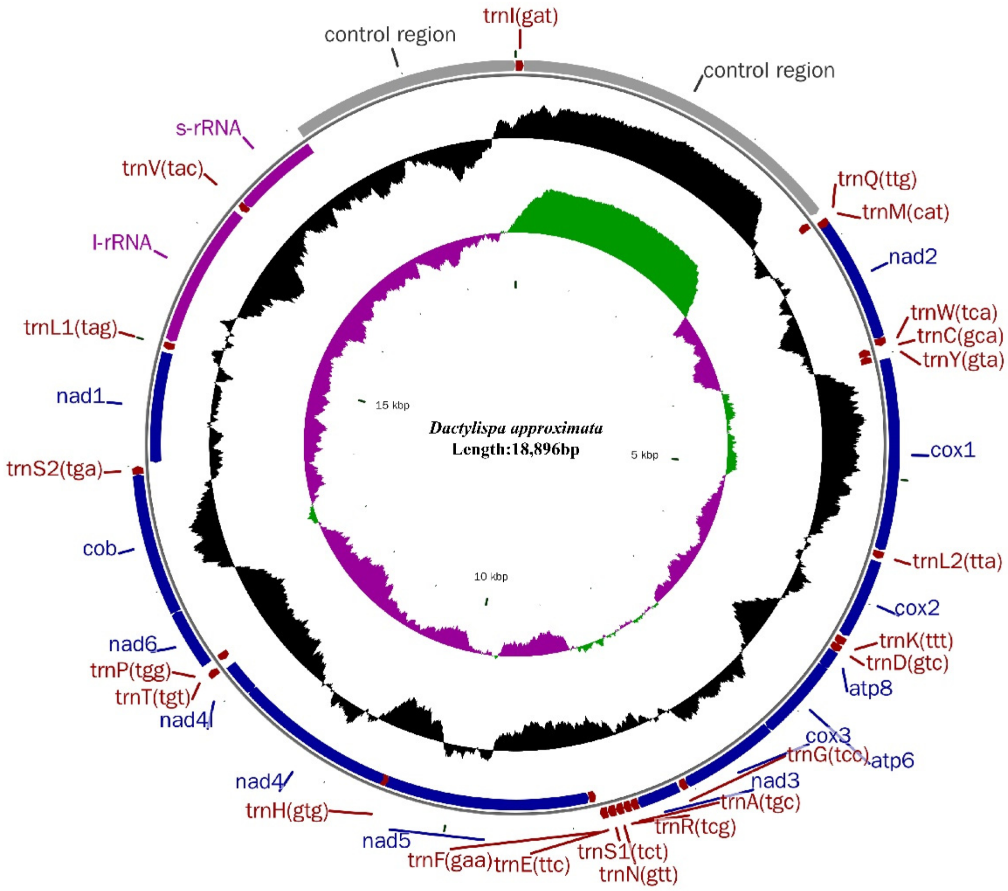

A

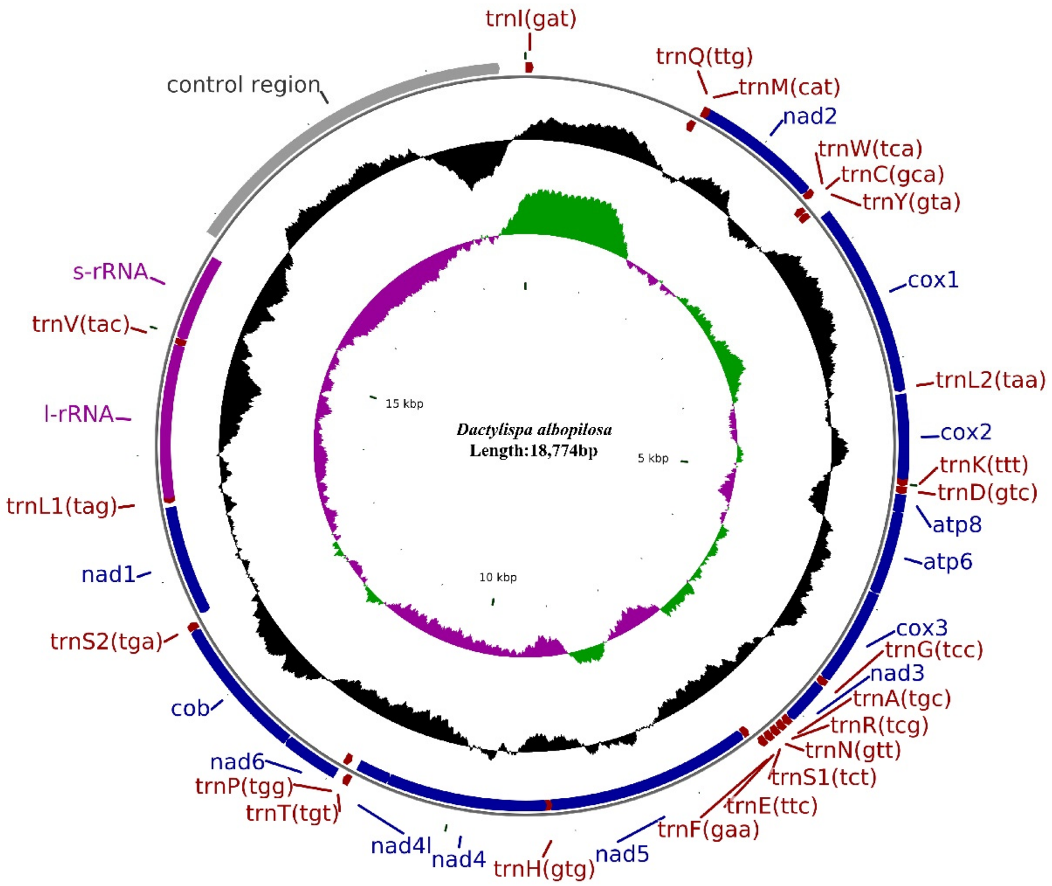

trnI(gat)
trnQ(ttg)
trnM(cat)
nad2
trnW(tca)
trnC(gca)
trnY(gta)
cox1
trnL2(taa)
cox2
trnK(ttt)
trnD(gtc)
atp8
atp6
cox3
trnG(tcc)
nad3
trnA(tgc)
trnR(tcg)
trnN(gtt)
trnS1(tct)
trnE(ttc)
trnF(gaa)
nad5
trnH(gtg)
nad4
nad4l
trnT(tgt)
trnP(tgg)
nad6
cob
trnS2(tga)
nad1
trnL1(tag)
l-rRNA
trnV(tac)
s-rRNA
control region
Dactylispa albopilosa
Length:18,774bp
5 kbp
10 kbp
15 kbp

B

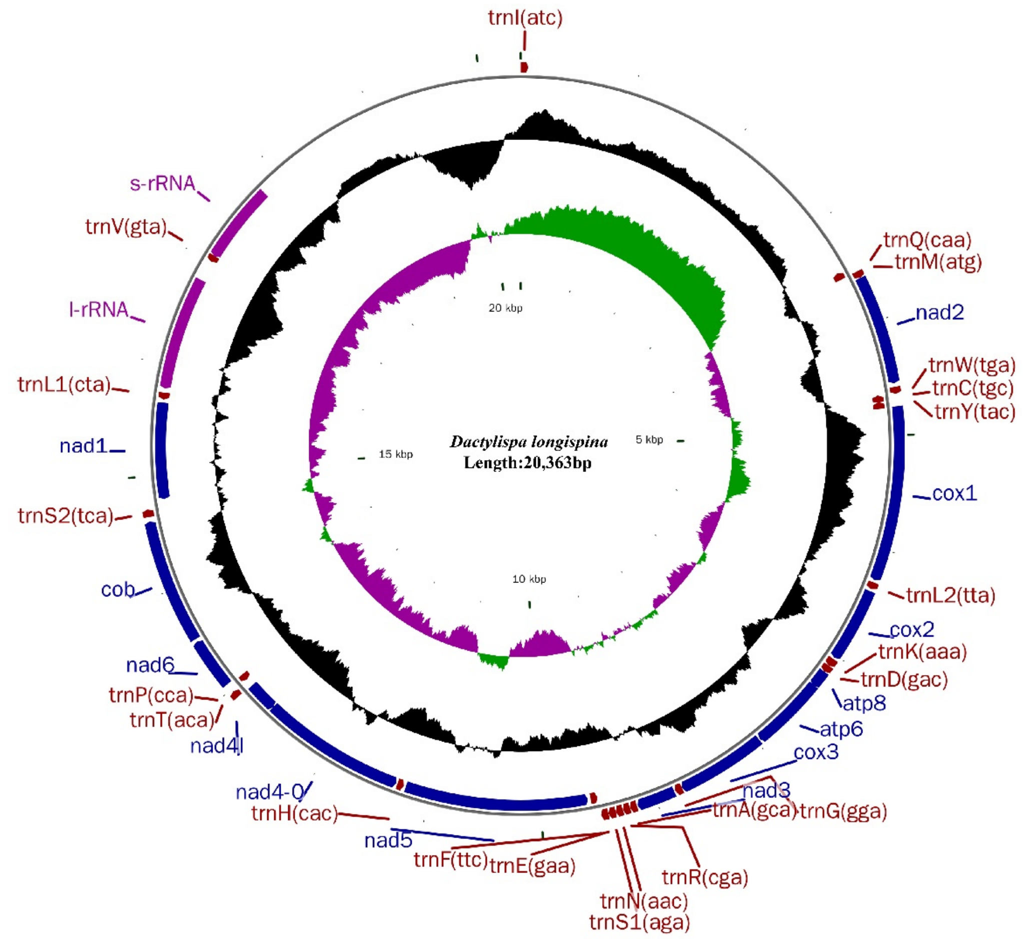
trnI(atc)
trnQ(caa)
trnM(atg)
nad2
trnW(tga)
trnC(tgc)
trnY(tac)
cox1
trnL2(tta)
cox2
trnK(aaa)
trnD(gac)
atp8
atp6
cox3
nad3
trnA(gca)
trnG(gga)
trnR(cga)
trnN(aac)
trnS1(aga)
trnE(gaa)
trnF(ttc)
nad5
trnH(cac)
nad4-0
nad4l
trnT(aca)
trnP(cca)
nad6
cob
trnS2(tca)
nad1
trnL1(cta)
l-rRNA
trnV(gta)
s-rRNA
Dactylispa longispina
Length:20,363bp
5 kbp
10 kbp
15 kbp
20 kbp

C

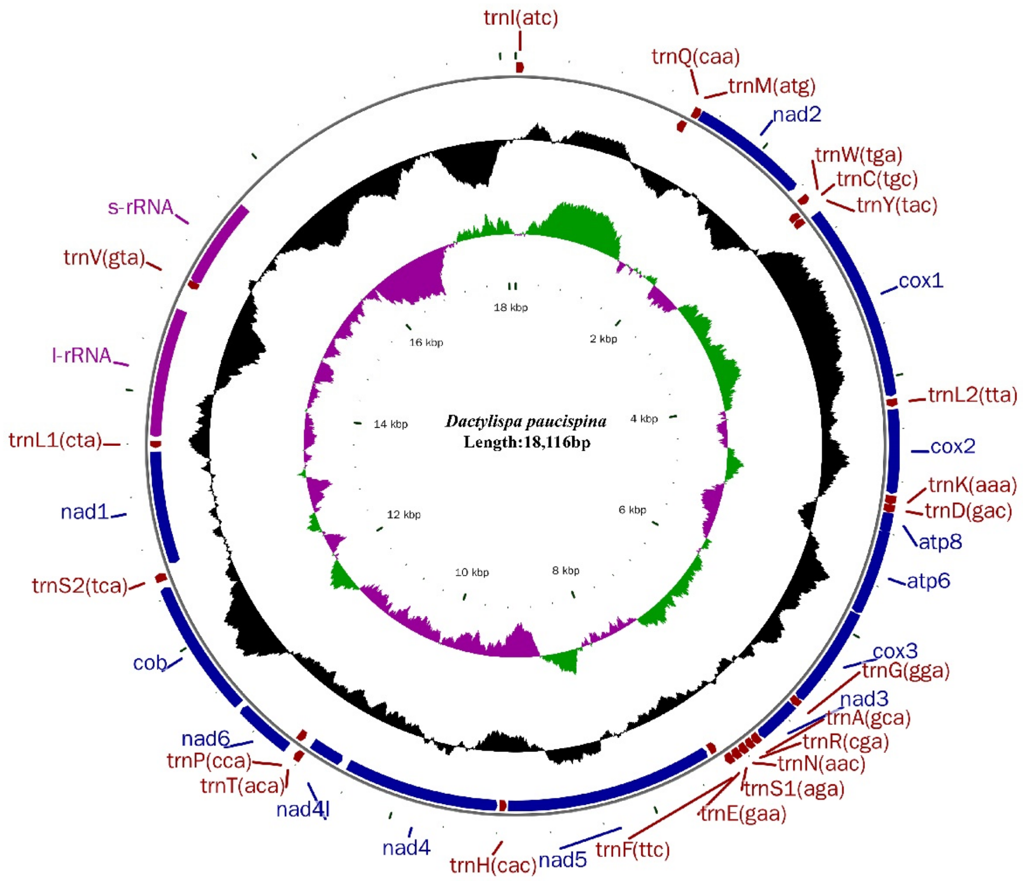
trnI(atc)
trnQ(caa)
trnM(atg)
nad2
trnW(tga)
trnC(tgc)
trnY(tac)
cox1
trnL2(tta)
cox2
trnK(aaa)
trnD(gac)
atp8
atp6
cox3
trnG(gga)
nad3
trnA(gca)
trnR(cga)
trnN(aac)
trnS1(aga)
trnE(gaa)
trnF(ttc)
nad5
trnH(cac)
nad4
nad4l
trnT(aca)
trnP(cca)
nad6
cob
trnS2(tca)
nad1
trnL1(cta)
l-rRNA
trnV(gta)
s-rRNA
18 kbp
2 kbp
4 kbp
6 kbp
8 kbp
10 kbp
12 kbp
14 kbp
16 kbp
Dactylispa paucispina
Length:18,116bp

D

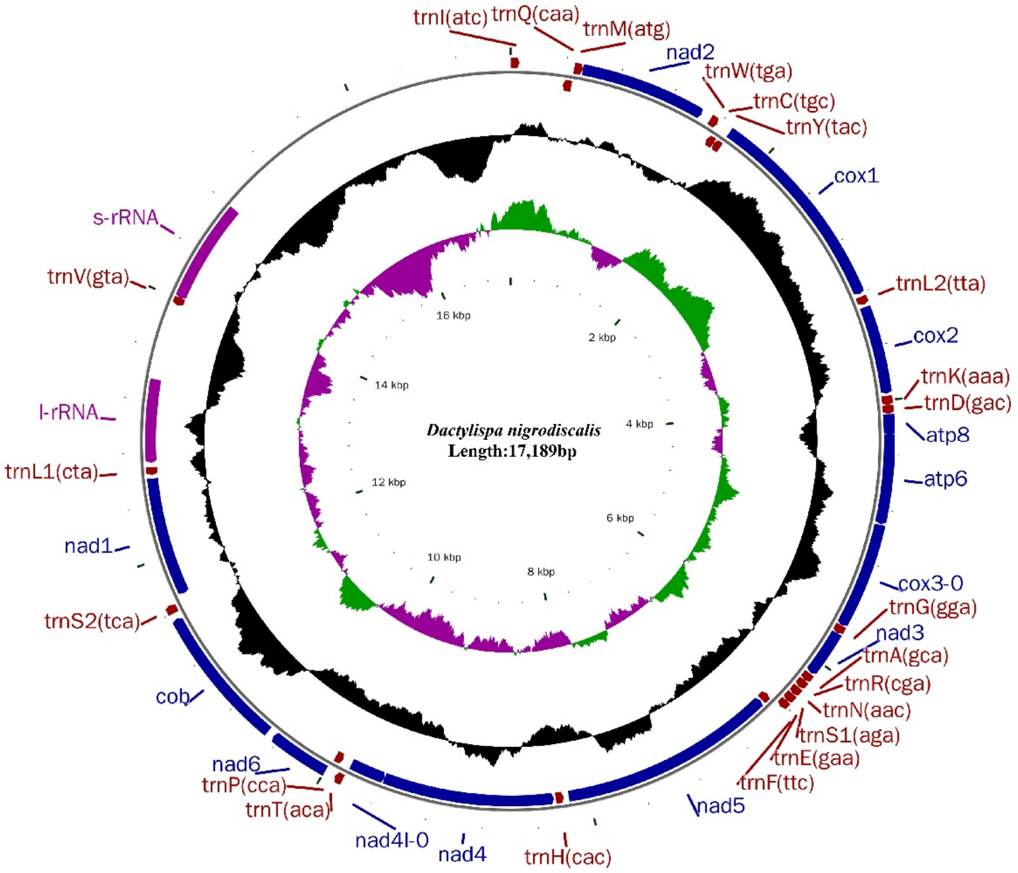
trnI(atc)
trnQ(caa)
trnM(atg)
nad2
trnW(tga)
trnC(tgc)
trnY(tac)
cox1
trnL2(tta)
cox2
trnK(aaa)
trnD(gac)
atp8
atp6
cox3-0
trnG(gga)
nad3
trnA(gca)
trnR(cga)
trnN(aac)
trnS1(aga)
trnE(gaa)
trnF(ttc)
nad5
trnH(cac)
nad4
nad4l-0
trnT(aca)
trnP(cca)
nad6
cob
trnS2(tca)
nad1
trnL1(cta)
l-rRNA
trnV(gta)
s-rRNA
Dactylispa nigrodiscalis
Length:17,189bp
2 kbp
4 kbp
6 kbp
8 kbp
10 kbp
12 kbp
14 kbp
16 kbp

E

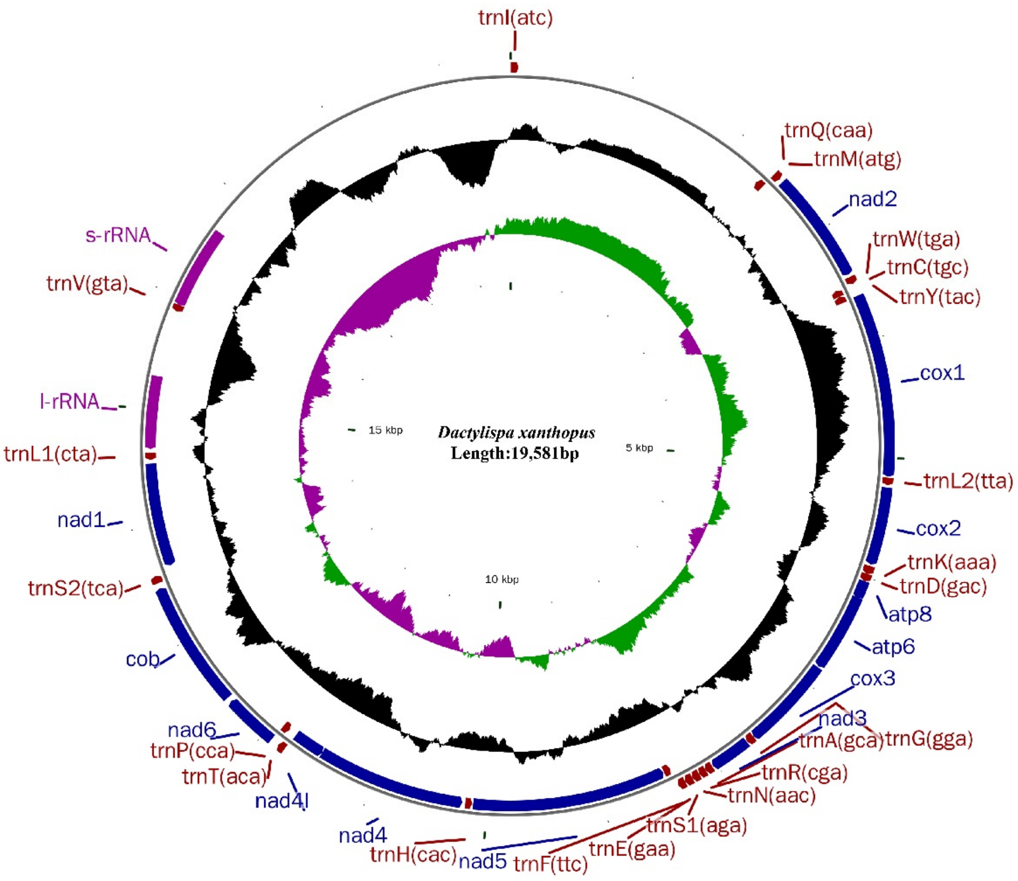
trnI(atc)
trnQ(caa)
trnM(atg)
nad2
trnW(tga)
trnC(tgc)
trnY(tac)
cox1
trnL2(tta)
cox2
trnK(aaa)
trnD(gac)
atp8
atp6
cox3
nad3
trnA(gca)
trnG(gga)
trnR(cga)
trnN(aac)
trnS1(aga)
trnE(gaa)
trnF(ttc)
nad5
trnH(cac)
nad4
nad4l
trnT(aca)
trnP(cca)
nad6
cob
trnS2(tca)
nad1
trnL1(cta)
l-rRNA
trnV(gta)
s-rRNA
Dactylispa xanthopus
Length:19,581bp
5 kbp
10 kbp
15 kbp

F

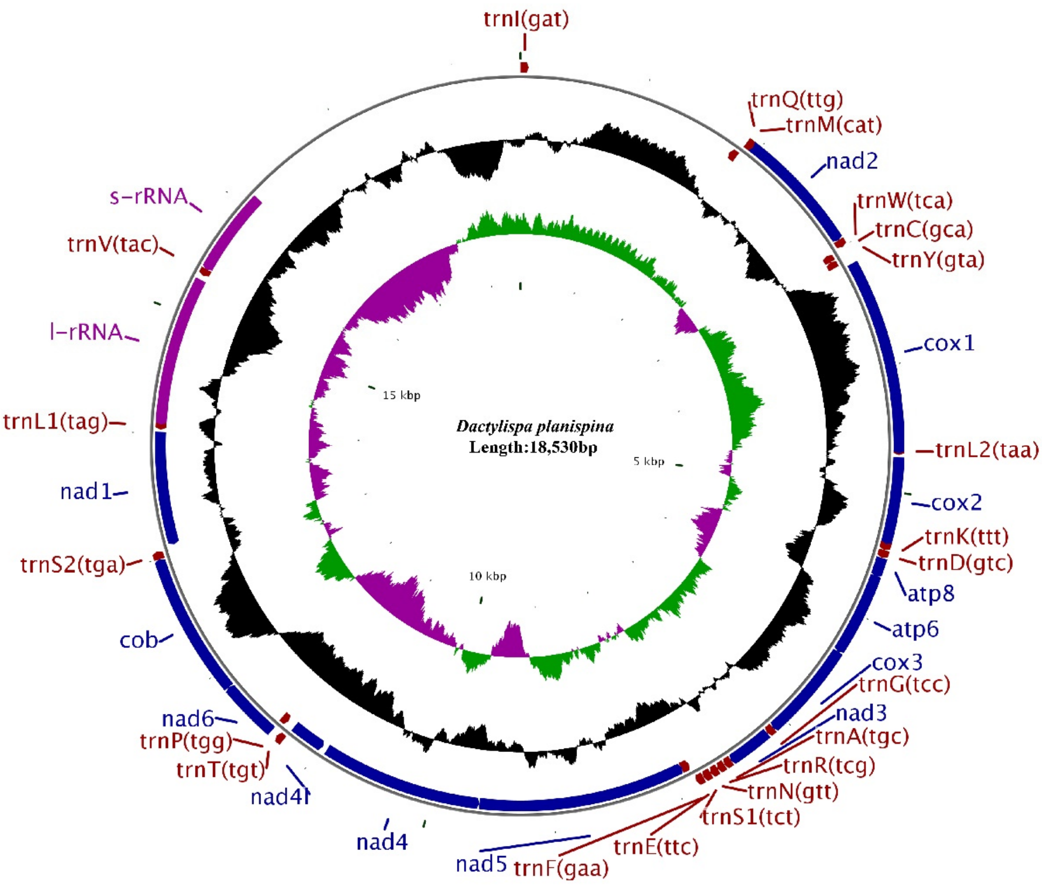
trnI(gat)
trnQ(ttg)
trnM(cat)
nad2
trnW(tca)
trnC(gca)
trnY(gta)
cox1
trnL2(taa)
cox2
trnK(ttt)
trnD(gtc)
atp8
atp6
cox3
trnG(tcc)
nad3
trnA(tgc)
trnR(tcg)
trnN(gtt)
trnS1(tct)
trnE(ttc)
trnF(gaa)
nad5
nad4
nad4l
trnT(tgt)
trnP(tgg)
nad6
cob
trnS2(tga)
nad1
trnL1(tag)
l-rRNA
trnV(tac)
s-rRNA
Dactylispa planispina
Length:18,530bp
5 kbp
10 kbp
15 kbp

G

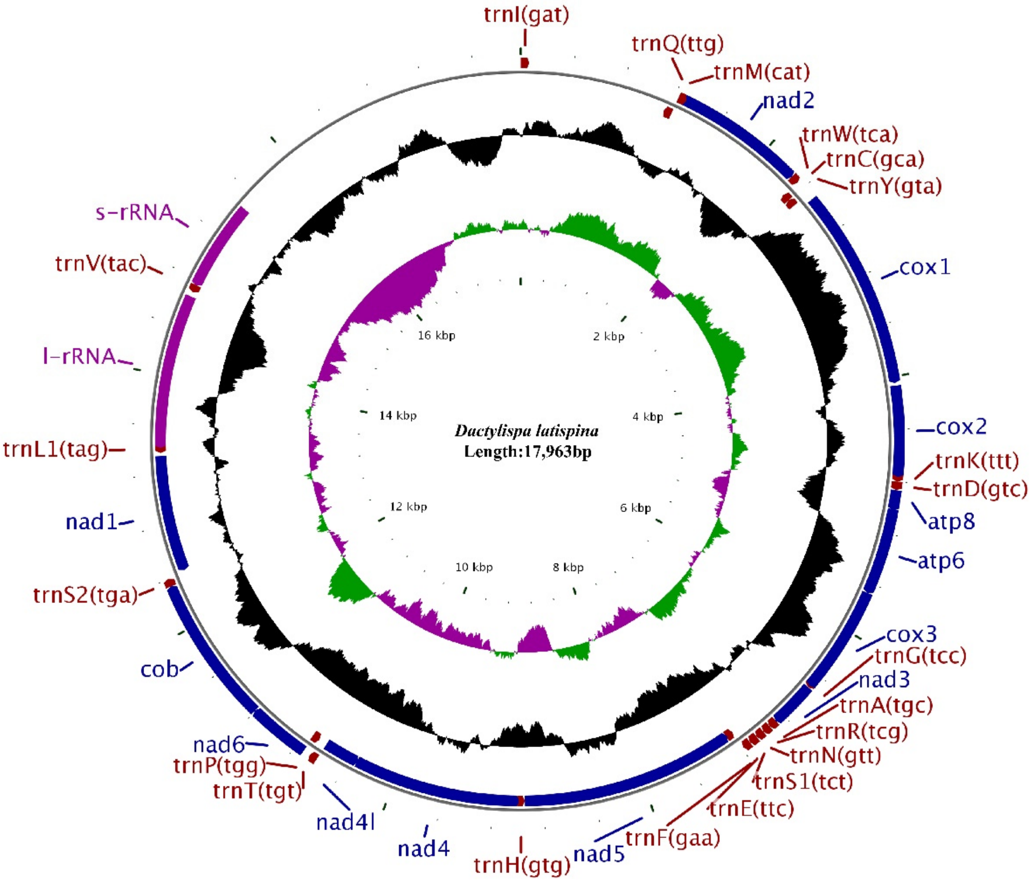
trnI(gat)
trnQ(ttg)
trnM(cat)
nad2
trnW(tca)
trnC(gca)
trnY(gta)
cox1
cox2
trnK(ttt)
trnD(gtc)
atp8
atp6
cox3
trnG(tcc)
nad3
trnA(tgc)
trnR(tcg)
trnN(gtt)
trnS1(tct)
trnE(ttc)
trnF(gaa)
nad5
trnH(gtg)
nad4
nad4l
trnT(tgt)
trnP(tgg)
nad6
cob
trnS2(tga)
nad1
trnL1(tag)
l-rRNA
trnV(tac)
s-rRNA
2 kbp
4 kbp
6 kbp
8 kbp
10 kbp
12 kbp
14 kbp
16 kbp
Dactylispa latispina
Length:17,963bp

H
